# Oxidative Stress-Mediated Apoptosis Induced by Ethanolic Mango Seed Extract in Cultured Estrogen Receptor Positive Breast Cancer MCF-7 Cells

**DOI:** 10.3390/ijms16023528

**Published:** 2015-02-05

**Authors:** Al-Shwyeh Hussah Abdullah, Abdulkarim Sabo Mohammed, Abdullah Rasedee, Mohamed Elwathig Saeed Mirghani

**Affiliations:** 1 Faculty of Food Science and Technology, Universiti Putra Malaysia, 43400 Serdang, Selangor, Malaysia; E-Mail: al-shwyeh-hussah@hotmail.com; 2 Faculty of Veterinary Medicine, Universiti Putra Malaysia, 43400 Serdang, Selangor, Malaysia; E-Mail: rasedee@upm.edu.my; 3 Institute of Bioscience, Universiti Putra Malaysia, 43400 Serdang, Selangor, Malaysia; 4 Department of Biotechnology Engineering Faculty of Engineering, International Islamic, University Malaysia (IIUM), 50728 Kuala Lumpur, Malaysia; E-Mail: elwathig@iium.edu.my

**Keywords:** MCF-7 cells, breast cancer, apoptosis, oxidative stress, mango kernel

## Abstract

Breast cancer has become a global health issue requiring huge expenditures for care and treatment of patients. There is a need to discover newer cost-effective alternatives for current therapeutic regimes. Mango kernel is a waste product with potential as a source of anti-cancer phytochemicals, especially since it is non-toxic towards normal breast cell lines at concentrations for which it induces cell death in breast cancer cells. In this study, the anti-cancer effect of mango kernel extract was determined on estrogen receptor-positive human breast carcinoma (MCF-7) cells. The MCF-7 cells were cultured and treated with 5, 10 and 50 μg/mL of mango kernel extract for 12 and 24 h. In response to treatment, there were time- and dose-dependent increases in oxidative stress markers and pro-apoptotic factors; Bcl-2-like protein 4 (BAX), p53, cytochrome c and caspases (7, 8 and 9) in the MCF-7 cells treated with the extract. At the same time, there were decreases in pro-survival markers (Bcl-2 and glutathione) as the result of the treatments. The changes induced in the MCF-7 cells by mango kernel extract treatment suggest that the extract can induce cancer cell apoptosis, likely via the activation of oxidative stress. These findings need to be evaluated further to determine whether mango kernel extract can be developed as an anti-breast cancer agent.

## 1. Introduction

Breast cancer is reported to cause the highest mortality among female cancer patients. Over a million women worldwide are diagnosed with breast cancer every year, and another 400,000 are reported to succumb to the disease [1,2,3]. The pathogenesis of cancers is multifactorial; however, oxidative stress is suggested to play a central role [4,5]. The balance of oxidative stress in cancers is critical, because it could either propagate cancers or induce death of cancer cells through apoptosis [6]. Apoptosis is a complex process involving multiple players, including pro-apoptotic factors, like the caspases (initiator caspases, caspase 2, 8, 9 and 10, and effector caspases, caspase 3, 6 and 7), Bcl-2-like protein 4 (BAX) p53 and cytochrome c, as well as pro-survival factors, like Bcl-2 and glutathione (GSH) [7,8]. Apoptosis is, in fact, the preferred mode of cell death in the treatment and control of cancers, because of its programmed nature and fewer sequelae. In recent years, apoptosis has received close attention as a potential mechanism that could be targeted through the activation of oxidative stress in cancer cells [7].

The treatment modality and prognosis of breast cancers are dependent on factors, such as metastases, tumor size, lymph node involvement, histological grade, estrogen (ER) and human epidermal growth factor type 2 and progesterone (PgR) receptors, as well as the proliferative marker, Ki67. Moreover, classification of breast cancer, especially its receptor status, is an important prelude to the management of the disease [9,10,11,12,13,14,15,16,17]. This is to ensure that the treatment options instituted provide the best prognosis. Estrogen-positive breast cancers grow faster in the presence of estrogen, and hence, anti-estrogenic drugs, like tamoxifen and aromatase inhibitors, are used to manage such cases [18,19,20].

In recent years, phytochemicals from plant bio-resources are being intensely investigated as better alternatives to the currently available therapies for breast cancers, due to their lesser side effects and costs of treatment. Polyphenols, for example, have been shown to have anti-breast cancer properties through the induction of apoptosis [21,22,23].

Mangoes, a fruit rich in phytochemicals, contain several bioactive compounds reported to have huge therapeutic potentials [24,25]. Thus, in this study, the effect of mango kernel extract was evaluated to determine its ability to induce the apoptosis of estrogen receptor-positive breast cancer cells.

## 2. Results and Discussion

### 2.1. Oxidative Stress Generation

**Figure 1:**
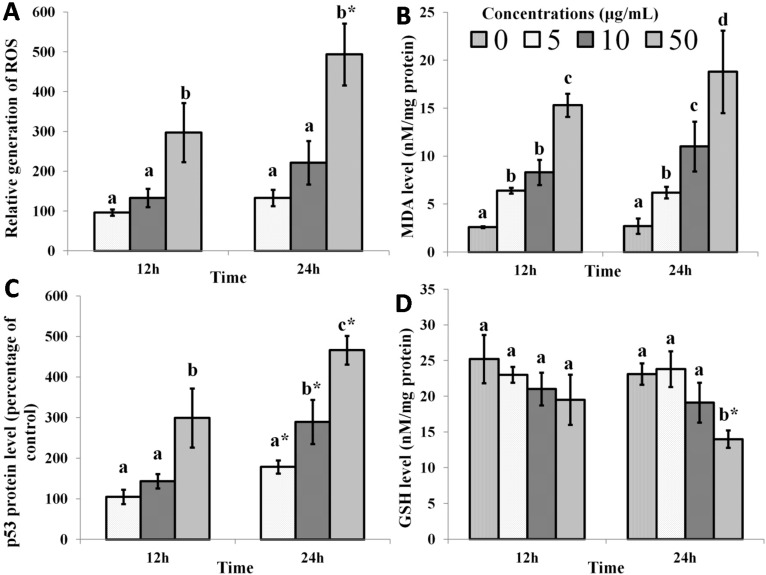
The effect of mango kernel extract on (**A**) the relative generation of reactive oxygen species (ROS) and (**B**) malondialdehyde (MDA) level; (**C**) p53 level and (**D**) Glutathione (GSH) level in MCF-7 cells. Treatment with mango kernel extract produced time- and dose-dependent changes in oxidative stress markers. Bars with different letters for any time period of an assay indicate a significant difference (*p* < 0.05). * Significant difference between 12 and 24 h with the same concentrations of extract.

Figure 1 shows the changes in oxidative stress markers in MCF-7 cells following treatment with mango extract for 12 and 24 h. Oxidative stress has been shown to induce apoptosis in cancer cells and has been indicated as a target for the anti-cancer effects of phytochemicals [26,27]. The results from this study showed an increase in reactive oxygen species (ROS) generation in a time- and dose-dependent manner in MCF-7 cells when treated with mango kernel extract (Figure 1A). This effect on ROS generation is mirrored by similar changes in malondialdehyde (MDA) levels, also in a time- and dose-dependent manner (Figure 1B). Since MDA, a product of lipid peroxidation, increases after extract treatment, this suggests that mango kernel extract promotes oxidative stress. This is supported by the decreasing trend of GSH concentration as a result of treatment with the extract. GSH is an endogenous antioxidant with multiple functions, including regulation of redox states in cells, and a decrease in its concentration may also be the cause of the development of oxidative stress in the MCF-7 cells. The decrease in the level of GSH upon treatment with mango kernel extract occurred in a dose-dependent manner over 24 h (Figure 1D).

Another method by which mango kernel extract promotes apoptosis is through stimulation of p53 activity. The tumor suppressor protein, p53, regulates growth and induces the apoptosis of tumorous cells, as well as mediating the removal of unwanted excessively dividing cells, thus preventing cancers. In cancers, reduction in p53 expression causes unfavorable loss of the regulation of cell growth [28,29]. The current work shows that mango kernel extract increased the level of p53 protein progressively over a 24 h period. This effect may be a consequence of the increase in oxidative stress induced in the cells by the extract.

### 2.2. BAX, Bcl-2 and Cytochrome c

**Figure 2:**
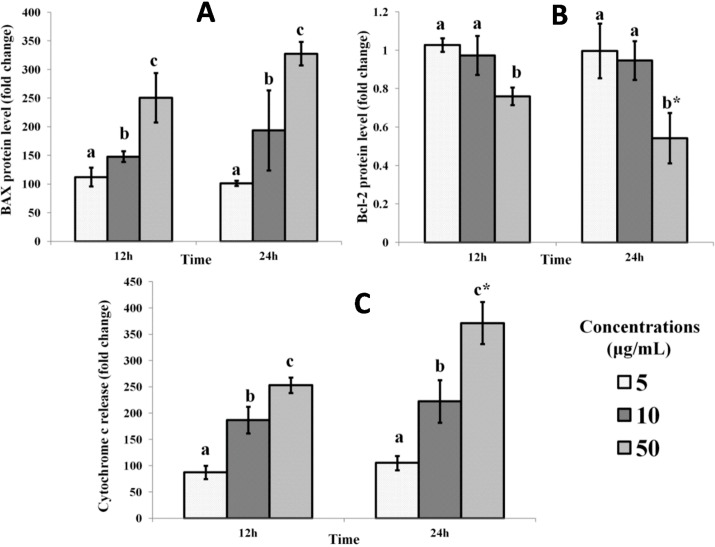
Effect of mango kernel extract on (**A**) BAX, (**B**) Bcl-2 and (**C**) cytochrome c levels in MCF-7. Treatment with 5, 10 and 50 μg/mL of extract for 12 and 24 h produced time- and dose-dependent changes in BAX, Bcl-2 and cytochrome c proteins. Bars with different letters for any time period of an assay indicate a significant difference (*p* < 0.05). * Significant difference between 12 and 24 h treatments with the same concentrations of extract.

Apoptosis is a complex process, which can be induced by oxidative stress, involving many factors, including BAX, Bcl-2 and cytochrome c as key regulators. In addition, cytochrome c release from the mitochondria in response to the induction of apoptosis effectively seals the fate of the cells by activating caspases, which then affect the apoptotic process [6,7,8]. In this study, there was a time- and dose-dependent increase in Bax and cytochrome c proteins, measured via the concentrations present in the cell culture medium, with a corresponding decrease in the level of Bcl-2 protein following treatment with mango kernel extract. This is evidence that the extract causes MCF-7 cell death via apoptosis (Figure 2). As suggested by the increase in ROS and MDA in this study, apoptosis of cancer cells induced by mango kernel extract is most likely the result of oxidative stress induction (Figure 1).

### 2.3. Caspases

**Figure 3:**
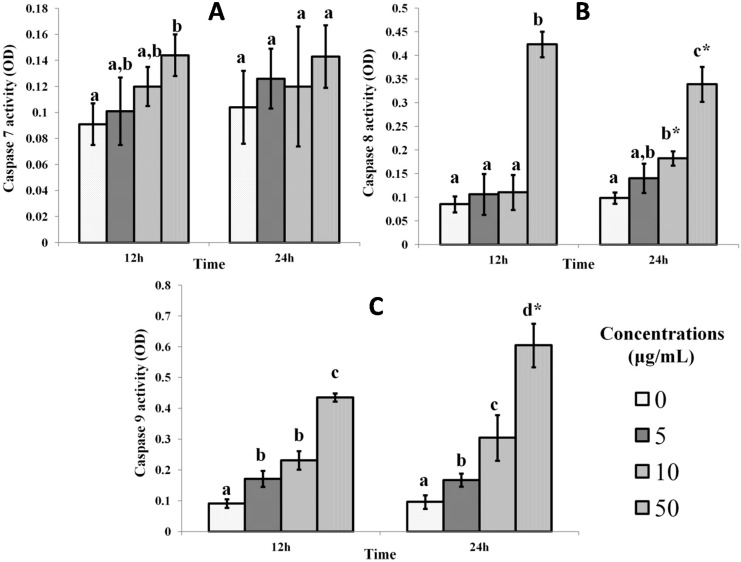
Effect of mango kernel extract on (**A**) caspases-3, (**B**) -8 and (**C**) -9 activities in MCF-7 cells. Treatment with 5, 10 and 50 μg/mL of extract over 12 and 24 h increased the levels of all caspases by 24 h, especially with the highest dose. Bars with different letters for any time period of an assay indicate a significant difference (*p* < 0.05). * Significant difference between 12 and 24 h treatments with the same concentrations of extract.

Figure 3 shows the effect of the mango kernel extract on caspases in MCF-7 cells. Mango kernel extract treatment had increased the activity of caspases 7, 8 and 9 after 24 h, with the highest dose (50 μg/mL) producing the greatest effect. During the treatment, cytochrome c was released, which activated caspases to effect apoptotic cell death through both the extrinsic (caspase 8) or intrinsic (caspase 9) pathways, mediated by an effector caspase, like caspase 7 [7].

In the study, the apoptosis of mango kernel extract-treated MCF-7 cells is believed to occur as the result of the upregulation of p53 proteins, which induced the expression of BAX and cytochrome c release from the mitochondria. Subsequent to these effects was the activation of the intrinsic and extrinsic pathways via caspases 7, 8 and 9 [28,29].

As reported recently, we have demonstrated that mango kernel extracts are non-toxic towards normal cells, but effective at inducing cell death in breast cancer cell lines [30]. Since this study showed that the extract is effective at killing cancer cells, it can potentially be developed into a safe therapeutic compound, especially for estrogen receptor-positive breast cancers.

## 3. Experimental Section

### 3.1. Preparation of Extract

Water lily mango fruits procured from a local market in Kuala Lumpur, Malaysia, during the period of June to July, 2012, were manually processed by soaking the kernels in water to remove the adhering flesh. The kernels were dried in an oven at 45 °C for 2 days, ground using Waring blender 7011HS (Osaka Chemical Co., Ltd., Kita-Ku, Osaka, Japan) and stored at 4 °C until analysis. Ethanol (95%) was added to the ground kernel at 10:1 (*v*/*w*), and the mixture was shaken at 200 rpm and 37 °C for 24 h in an incubator shaker (INNOVA 4000, Edison, NJ, USA). Insoluble materials were removed by filtration, and the solution was centrifuged at 4000 rpm (Benchtop Centrifuge Z200A, Labnet International, Inc., Woodbridge, NJ, USA) for 10 min to obtain the supernatant. The supernatant was dried using the 1 L Rotary Evaporator N1001S-WD (Tokyo Rikakikai Co., Ltd., Koishikawa Bunkyo-ku, Tokyo, Japan), and the extract was finally dissolved in dimethylsulfoxide (DMSO) and kept at −20 °C until analysis.

### 3.2. Cancer Cell Lines

Human breast carcinoma (MCF-7) cell lines are often used *in vitro* studies of breast cancers [31]. The MCF-7 cells were obtained from the American Type Culture Collection (ATCC, Rockville, MD, USA) and cultured in Dulbecco’s modified eagle’s medium (DMEM) supplemented with 10% fetal bovine serum (FBS) and 1% antibiotics (100 U/mL penicillin) in an incubator at 37 °C under 5% CO_2_.

### 3.3. Determination of ROS, Thiobarbituric Acid Reactive Substances and GSH

ROS generation was determined in the MCF-7 cells after treatment with 5, 10 and 50 μg/mL mango kernel extracts for 12 and 24 h [32]. Briefly, treated cells were washed with phosphate-buffered saline containing 2',7'-dichlorofluorescein diacetate to induce oxidation of 2',7'-dichlorofluorescein diacetate to dichlorofluorescein by ROS. The cells were then lysed in buffer (50 mM Tris-HCl, 100 mM NaCl, 1 mM CaCl, 1 mM MgCl, 300 mM sucrose, 1% Triton X-100, pH 7.4), and the fluorescence of suspensions was determined in a quartz cuvette at 530 nm with an excitation wavelength of 485 nm. Treated cells were then washed with phosphate-buffered saline (PBS), harvested, homogenized in ice-cold 1.15% KCl and then subjected to the thiobarbituric acid reactive substances (TBARS) assay to determine the development of oxidative stress [32]. The TBARS are products generated by lipid peroxidation. Results were expressed as MDA equivalent (nM/mg protein).

GSH assays were also performed on treated MCF-7 cells [32]. Results were determined colorimetrically at 405 nm using a UV-vis spectrophotometer, and expressed as nM/mg of cell lysate protein.

### 3.4. Bcl-2, Bax, p53 and Cytochrome c Protein Assays

Bax, Bcl-2, p53 and cytochrome c proteins were assayed using enzyme-linked immune-sorbent assay kits (R&D Systems, Minneapolis, MN, USA), as reported previously [32,33]. Briefly, the protein assays were conducted on abstract-treated cells according to the manufacturer’s instructions. The absorbance was read on a micro-plate reader, and the results are expressed as the fold change in protein expression (relative expression in treated against untreated cells).

### 3.5. Caspase-7, -8 and -9 Activities

Caspase-3/7, -8 and -9 activities were determined on extract-treated cells using commercial kits (Promega, Madison, WI, USA), according to the manufacturer’s instructions [33]. Briefly, after treatment, the cells were harvested by centrifugation. The pellets were washed with PBS before lysis in chilled lyses buffer. The mixture was then left on ice for 10 min and centrifuged at 50× *g* (Eppendorf Centrifuge 5810R, Hamburg, Germany) at 4 °C for 5 min. The resultant supernatant was used for the determination of caspase activity. The results were read on a micro-plate reader at 405 nm.

### 3.6. Statistical Analysis

All experiments were performed in triplicate, and the data were expressed as the mean ± standard deviation. The data were analyzed using Minitab statistical software (Minitab Inc., State College, PA, USA). One-way analysis of variance and Tukey’s *post hoc* analysis was done to determine statistical significance (*p* < 0.05).

## 4. Conclusions

The study showed that mango kernel extract increases apoptotic markers in MCF-7 cells, which likely resulted from increased oxidative stress. Apoptosis of MCF-7 cells was mediated by the increase in pro-apoptotic factors, including Bax, cytochrome c, p53 and caspases, and a reduction in pro-survival factors, GSH and Bcl-2. These findings suggest that mango kernel extract has potential to be developed as an anti-breast cancer agent.
